# Novel micellar CB2 receptor agonist with anti-inflammatory action for treating corneal alkali burns in a mouse model

**DOI:** 10.3389/fphar.2023.1270699

**Published:** 2023-12-15

**Authors:** Neethi C. Thathapudi, Marc Groleau, Delali S. Degué, Mozhgan Aghajanzadeh Kiyaseh, Piotr Kujawa, Fouzia Soulhi, Naoufal Akla, May Griffith, Marie-Claude Robert

**Affiliations:** ^1^ Maisonneuve-Rosemont Hospital Research Centre, Montreal, QC, Canada; ^2^ Department of Ophthalmology, Université de Montréal, Montreal, QC, Canada; ^3^ Institute of Biomedical Engineering, Université de Montréal, Montreal, QC, Canada; ^4^ Department of Microbiology, Infectiology and Immunology, Université de Montréal, Montreal, QC, Canada; ^5^ Pharmaceutical Research and Development, Altus Formulation Inc., Laval, QC, Canada

**Keywords:** cornea, anti-inflammatory, alkali burn, mouse, steroid, CB2r

## Abstract

**Introduction:** Moderate corneal alkali burns such as those sustained from accidental exposure to household chemicals are treated with topical corticosteroids. Side effects include increased intraocular pressure and slowing of wound healing. Here, we compare the effects of a cannabinoid receptor 2 (CB2r) agonist, TA-A001, that is involved in wound healing with that of the corticosteroid, prednisolone.

**Methods:** TA-A001 was encapsulated with a polymeric micelle comprising polyvinylpyrrolidone: polylactide block copolymers referred to as SmartCelle™ to allow delivery of the very hydrophobic drug. Mouse corneas were given moderate alkali burns. Different doses of TA-A001 of 0.125%, 0.25% and 0.5% were used to treat the burns in comparison to the corticosteroid, prednisolone.

**Results:** TA-A001 at 0.25% and 0.5% allowed for faster wound closure. However, the higher 0.5% dose also induced unwanted neovascularization. By comparison, burned corneas treated with prednisolone showed slower healing as well as disorganization of the cornea. Although 0.25% TA-A001 appeared to produce the most-optimal responses, this dose resulted in marked expression of the macrophage chemoattractant protein, MCP-1. However, there was also an increase in CD163 positive stained M2 anti-inflammatory macrophages in the TA-A001 corneas. TA-A001 treated corneas showed the presence of sensory nerve fibers throughout the corneal epithelium including the superficial cell layers as did Substance P staining.

**Discussion:** We found that TA-A001 at the 0.25% doses was able to modulate inflammation resulting from a moderate alkali burn to the cornea. With more extensive testing, TA-A001 might prove to be a potential alternative to corticosteroids for treating alkali burns or other causes of corneal inflammation.

## Introduction

Topical corticosteroids are routinely used to control inflammation following corneal alkali burn as well as many other corneal diseases (Donshik et al., 1978). Corticosteroids function by binding to glucocorticoid receptors that modulate over 5000 genes and exert immunosuppressant effects (Comstock and Decory, 2012). However, despite their widespread use, they have many notable side effects. The more concerning side effects are glaucoma, cataracts, and enhancement of infectious diseases. In the case of glaucoma, corticosteroids can impact the trabecular meshwork by increasing the amount of extracellular material deposited that in turn blocks the aqueous outflow (Kersey and Broadway, 2006). With reduced outflow, the intraocular pressure (IOP) can build up, putting stress onto the optic nerve, resulting axonal damage, constriction of the visual field and ultimately, loss of vision. For cataracts, it is unclear how corticosteroids affect the lens. There is speculation that steroids may bind directly to the lens and their accumulation leads to decreased clarity and eventually cataracts (Manabe et al., 1984; Dickerson et al., 1997). Recent evidence suggests that corticosteroids cause apoptosis and prevent lens epithelial cells from differentiating fully (Jobling and Augusteyn, 2002; Petersen et al., 2008). The enhancement of infectious diseases by corticosteroids can be attributed to decreased wound healing and blunted immune response. Corticosteroids can also induce the reactivation of latent viruses such as Herpes Simplex type 1, although the mechanism is still unclear (Gulkilik et al., 2007; Jusufbegovic and Schaal, 2017). Corticosteroid treatment can delay the healing of corneal epithelial defects in rabbits (Sarchahi et al., 2011) and may also promote thinning of the corneal stroma (referred to as sterile corneal melt) following alkali burn due to decreased collagen synthesis.

Interesting alternatives to corticosteroids are the agonists to cannabinoid receptors 1 and 2 (CB1r and CB2r). CB1r and CB2r are common in the human cornea, limbus, and conjunctiva and have been shown to play a role in wound healing (Straiker et al., 1999; Iribarne et al., 2008). In a murine dry eye disease model, treatment with CB1r and CB2r agonists resulted in better corneal health, with increased nerve fiber length and decreased T cell infiltration compared to the non-treated eyes (Steven et al., 2019). In a mouse model, less immune cell infiltration within the stroma and less scarring compared to knockout mice lacking corneal cannabinoid receptors, was observed (Yang et al., 2013). Additionally, cannabinoids can reduce IOP by promoting the aqueous outflow, with a lower IOP reducing the risks of developing glaucoma (Cairns et al., 2016; Thapa et al., 2018). One way it does this is by upregulating enzymes that degrade extracellular matrix in the trabecular meshwork (Panahi et al., 2017; Weinreb et al., 2020). Additionally, there is some evidence that CB1r and CB2r agonists, in addition to 5-HT1A, can reduce pain as observed in a mouse study where treated mice blinked, squinted, and rubbed their eyes less frequently (Thapa et al., 2018). Adverse events from ocular cannabinoid treatments are not yet fully understood. In rabbit corneas, it was observed that CB1r signaling promoted corneal neovascularization, while reduced neovascularization was observed when CB1r activity was blocked (Pisanti et al., 2011). While neovascularization is often associated with inflammation, many studies report a reduction of inflammation with CB1r activation (Straiker et al., 1999; Iribarne et al., 2008; Yang et al., 2013; Cairns et al., 2016; Panahi et al., 2017; Thapa et al., 2018; Murataeva et al., 2019; Steven et al., 2019; Weinreb et al., 2020). Additionally, the study reporting on neovascularization did not examine inflammatory markers beyond indicators for neovascularization (Pisanti et al., 2011). Taken together, CB1r and CB2r are very promising routes to suppress inflammation, especially CB2r which plays an important role in wound healing.

[(1R,2R,5R)-2-[2,6-dimethoxy-4-(2-methyloctan-2-yl)phenyl]-7,7-dimethyl-4-bicyclo[3.1.1]hept-3-enyl] methanol or TA-A001 is a proprietary low molecular weight small molecule that has both analgesic and anti-inflammatory activities. TA-A001 is an endocannabinoid receptor agonist and displays CB2r endocannabinoid receptor activity via a specific ratio of two enantiomers (E1 and E2) [Patent (Smith et al., 2022a)]. TA-A001 is highly insoluble in aqueous solutions (<0.5 mg/L) and therefore its use as a drug necessitates delivery with a solubility enhancing excipient. A polyvinylpyrrolidone: polylactide block copolymer excipient SmartCelle™ was therefore developed as a micellar delivery system for the delivery of TA-A001.

Our objective was to examine the efficacy of micelle-encapsulated TA-A001 or micellar TA-A001 in corneal wound healing after a moderate alkali burn, such as from accidental exposure to household cleaning items, in a mouse model. We evaluated its performance against prednisolone, a commonly used corticosteroid in the treatment of burns as other common causes of corneal inflammation.

## Materials and methods

### Preparation of micellar TA-A001

TA-A001 [Patent (Smith et al., 2022a)] was purchased from Tocris Bioscience (Toronto, ON, Canada). The small molecule was characterized on its own and in micellar form using a qualified HPLC method. The system comprised a Agilent HPLC, model 1100 equipped with a variable wave-length detector (VWD) or diode array detector (DAD) set at 210 nm, a Zobax Eclipse XD8-C8 column (3.5 mm 150 × 4.6 mm) and a water:acetonitrile gradient mobile phase. System suitability tests performed for each analytical run showed a percentage recovery of 99%–101%. Relative standard deviation (SD) of standards and quality control samples assay were <2%. All assays were performed in duplicate.

Poly(N-vinyl-2-pyrrolidone)-block-poly(D,L -lactide) (PVP-PLA) copolymer (SmartCelle) were prepared as previously described [Patent (Smith et al., 2022a; Smith et al., 2022b)], see Supplementary Material for details. SmartCelle TA-A001 formulation was prepared as described in Smith et al. (2022a). Briefly, SmartCelle was dissolved in ethanol. TA-A001 at a drug loading of 2% was subsequently added and kept under stirring until a clear solution was obtained. Purified water was added to the solution to obtain a final TA-A001 concentration of 1 mg/mL. The Smartcelle TA-A001 mixture was concentrated to 10% of its initial weight using a Rocket synergy evaporator (Thermofisher Scientific). Phosphate buffer saline (PBS) was then added to the concentrated solution to obtain final concentration of 4 g/L. The bulk solution was then filtered using 0.2 μm filter and transferred to 10 mL glass vial and lyophilized in a VirTis Genesis 25 EL lyophilizer. The lyophilized cake was reconstituted at 0.125, 0.25% and 0.5% by varying the amount of water for injection. The vehicle control comprised SmartCelle solution only. The average pH of the reconstituted solution was 7.4 ± 0.1 as measured using a pH211 pH-meter (Hanna instrument) equipped with gel-filled epoxy-body combination electrode. The size (Z-average) of the micelles were determined at 25°C by dynamic light scattering using a Malvern Nano ZS90 Zetasizer with 90° scattering optics equipped with 4 mW He-Ne laser operating at 633 nm.

### *In Vitro* bone marrow-derived dendritic cell (BMDC) culture and flow cytometric analysis

With ethical permission from the Animal Care and Use Committee of Maisonneuve-Rosemont Hospital (protocol #2023-3239), bone marrow was obtained from the tibia and femur of male C57BL/6J mice (6–12 weeks old). Bone marrow cells were divided into 10^6^ cells per well in a suspension culture plate and were cultured in RPMI 1640 containing 10% (v/v) fetal bovine serum (FBS) (Wisent, St-Bruno, QC, Canada), penicillin-streptomycin-glutamine (0.5 mg/mL), 10 mM Hepes, 1 mM sodium pyruvate, 55 μM of β-mercaptoethanol, and granulocyte-macrophage colony-stimulating factor (2.5 ng/mL; GM-CSF) (All from Gibco, Thermo Fisher Scientific, Waltham, MA, United States). After 2 and 3 days from the initial seeding, half the media was exchanged for fresh media containing a higher concentration of GM-CSF (5.0 ng/mL). On day 6, the suspended cells were collected and a Histodenz density gradient (Sigma-Aldrich, St. Louis, MO, United States) was used to isolate enlarged cells. A million cells were added to a 24-well suspension plate containing the following treatment and 2 mL of media.

Cells were exposed to either 45 µL 0.5% CB2r agonist formulation (TA-A001), 45 µL SmartCelle delivery vehicle, 45 µL prednisolone, 1 μg/mL LPS or no treatment by adding the components individually to the cell culture medium. After the treatment was added, the cells were cultured for an additional 24 h. At 18 h, GolgiStop (Becton, Dickinson and Company, Franklin Lakes, NJ, United States) was added to prevent the secretion of cytokines. Cells were stained for surface markers CD11c, CD40, CD80, and CD86 (Table 1) and Zombie Aqua Fixable Viability Kit (BioLegend, San Diego, CA). Fixation and permeabilization (Becton, Dickinson and Company, Franklin Lakes, NJ, United States) was performed to allow for intracellular staining of TNF-α (Table 1). A BD LSR II flow cytometer was used on all the samples and were analyzed using FlowJo software (Becton, Dickinson and Company, Franklin Lakes, NJ, United States). All cell suspensions were sampled and analyzed for the same duration and speed with an LSR II. BMDCs were selected by CD11c expression and live Zombie Aqua staining. Mean fluorescence intensity (MFI) of TNF-α, CD40, CD80, and CD86 of treated cells were compared to untreated cells to form a ratio.

**TABLE 1:** Antibodies for flow cytometry.

Target	Antibody	Dilution factor
CD11c	Brilliant Violet 650™ anti-mouse CD11c, (Clone: N418), (IsoType: Armenian Hamster IgG), (Reactivity: Mouse), (Format: BV650), (APP: FC), (Species: Hamster), Biolegend, 117339	1:1600
TNF-a	PerCP/Cyanine5.5 anti-mouse TNF-α, (Clone: MP6-XT22), (IstoType: Rat IgG1, κ), (Reactivity: Mouse), (Format: PerCP/Cyanine5.5), (APP: FC), (Species: Rat), Biolegend, 506321	1:800
CD40	CD40, APC, clone: 1C10, eBioscience™, 501129392	1:400
CD80	PE anti-mouse CD80, (Clone: 16-10A1), (IsoType: Armenian Hamster IgG), (Reactivity: Mouse, Cross-Reactivity: Dog (Canine)), (Format: PE), (APP: FC), (Species: Hamster), Biolegend, 104708	1:1600
CD86	FITC anti-mouse CD86, (Clone: GL-1), (IsoType: Rat IgG2a, κ), (Reactivity: Mouse), (Format: FITC), (APP: FC), (Species: Rat), Biolegend, 105006	1:50

### Animals and treatments

All procedures were conducted in accordance to the guidelines of the Association of Research in Vision and Ophthalmology statement for animal use. Ethical approval was obtained from the animal care committee of the Maisonneuve-Rosemont Hospital Research Centre (protocol number #2021-2356) prior to the start of the study. Six-to ten-week-old female BALB/c mice were housed under a photoperiod of 12:12 light/dark cycle with a room temperature between 21°C and 24°C. Females were selected as there were no reported differences in healing outcomes on alkali burns between the sexes, and female animals are more docile for handling and can be housed together. The mice were divided randomly into five experimental groups of 10 animals each by the animal care staff on arrival to the facility with five animals per cage. After full anesthesia, induced by isoflurane gas, 0.5% tetracaine eyedrops were applied to the cornea and corneal alkali burns were performed in the anesthetized eyes. A 2 mm diameter Whatman no.1 filter paper soaked in 0.25N sodium hydroxide (NaOH) was applied centrally to the right cornea (OD) for 15 s. The eyes were immediately rinsed with 0.01 M PBS to remove any excess NaOH. Each group of mice were then treated with the delivery vehicle, SmartCelle micelles, containing 0, 0.125, 0.25 or 0.5% TA-A001. Treatment with a corticosteroid, Teva-Prednisolone 1% (DIN. 00700401), served as a benchmark. Each group also had 3 mice that did not receive the alkali burn but only received the treatment, serving as controls to ensure that the drugs did not adversely affect normal, healthy corneas.

All treatments were given as 15 μL eye drops 3 times daily for 2 weeks, with a gap of 3 hours between treatments. Additionally, all mice received 4 days of Tobradex (tobramycin 3 mg/mL—dexamethasone 1 mg/mL suspension) (DIN. 00778907) followed by 3 days of Tobramycin 3 mg/mL antibiotic drops (DIN.02241755) on their burned eye twice daily. Mice in the Prednisolone group were given an additional analgesic Buprenorphine 0.05 mg/kg, on days 2, 3 and 4 post-burn after showing symptoms of pain such as closed eyes, poor grooming and decreased activity.

### Clinical examinations

Eye examinations that included esthesiometry, tonometry, and optical coherence tomography (OCT) were performed the day before the burn and at 2 weeks post burn for both eyes. Body weights were measured pre-operatively and at every 2 days to assess the overall health of the animal. The overall level of grooming and activity of the mice were noted as additional indicators of wellbeing.

Eye exams were conducted on mice under anesthesia with isoflurane. The overall clarity, redness and presence of any discharge was noted. Aesthesiometry was performed for nerve touch sensitivity on the corneas using a Cochet-Bonnet aesthesiometer (Handaya Co., Tokyo, Japan) before anesthesia. Intraocular pressure (IOP) measurements were performed on anesthetized mice, using an iCare Tonolab tonometer (Icare Finland Oy, Vantaa, Finland). Optical Coherence tomography (OCT) was performed using a portable instrument (Lumedica OQ Labscope 2.0 Imaging System, Edmund Optics, Barrington, NJ) to determine corneal health in live animals, under anaesthesia. Total corneal thickness was measured from the OCT images using Fiji software. Sodium fluorescein staining was performed daily throughout the 14-day observation period to determine the rate of epithelial wound closure. Quantification was performed on images taken during the period of treatments, using Fiji software. The image was split into its RBG file and the green channel image was used for the quantification. The burned area was quantified as a percentage area within the cornea. The eyes were collected and the corneas were either processed as flat-mounts, or embedded and cryosectioned for immunohistochemistry.

### Flat-mount immunohistochemistry

For flat mount staining of CD31 neovascularization, excised eyes were fixed in 2% PFA in 0.01 M PBS at 4°C, overnight, and then stored in 0.01 M PBS until they were stained. Eyes were dissected into corneoscleral buttons. Corneas were blocked using a solution containing 5% normal goat serum, 0.5% Triton X-100 in 0.01 M PBS for 2 hours. Antibodies were diluted in the blocking solution, and each were incubated with the samples with washing in between the primary and secondary antibodies (Table 2). Corneas were then flattened onto a microscopy slide and immersed with Fluoroshield mounting medium containing DAPI (Sigma-Aldrich, St. Louis, MO, United States). All samples were imaged as a tilled z-stack on a Zeiss Axio Imager Z2 with an AxioCam MRc color CCD camera (Carl Zeiss, Oberkochen, Germany). Images were compressed into a maximum projection using extended depth of focus and stitched based on the vessel staining in Zen Blue. The distance between the large collecting vessels and the furthest vessel was measured in FIJI. Measurements were grouped into four categories. 0 = <300 µm, 1 = 300–400 µm, 2 = 401–500 µm, 3 = >500 µm.

**TABLE 2:** Antibodies used for immunohistochemistry.

Target	Antibody	Dilution factor
F4/80	F4/80 Antibody (C-7), Santa Cruz, SC-377009	1:200
CD163	Thermo Fisher Scientific CD163 Monoclonal Antibody, Invitrogen, 14163182	1:200
LYVE1	Recombinant Anti-LYVE1 antibody [EPR21771] Abcam, ab218535	1:500
Muc1	MUC1 Antibody—BSA Free, Novus Biologicals	1:100
CD63	Anti-CD63 Antibody (MX-49.129.5), Santa Cruz, sc-5275	1:1000
TSG101	Recombinant Anti-TSG101 antibody [EPR7130(B)], AbCam, ab125011	1:1000
Monocyte chemoattractant protein-1	Ultra-LEAF™ Purified anti-mouse/rat/human MCP-1 Antibody, BioLegend, 505911	1:500
βIII Tubulin	Anti-beta III Tubulin antibody - Neuronal Marker, Abcam, ab18207	1:200
Substance P	Anti-Substance P Antibody, pain, clone NC1, Millipore Sigma, MAB356	1:200
CD31	Purified Rat Anti-Mouse CD31, BD Biosciences, 553370	1:100
Mouse IgG	Goat anti-Mouse IgG (H + L) Highly Cross-Adsorbed Secondary Antibody, Alexa Fluor™ Plus 647, Invitrogen, A32728	1:1000
Rabbit IgG	Goat anti-Rabbit IgG (H + L) Highly Cross-Adsorbed Secondary Antibody, Alexa Fluor™ 488, Invitrogen, A11034	1:1000
Rabbit IgG	IgG (H + L) Highly Cross-Adsorbed Goat anti-Rabbit, Alexa Fluor™ 594, Invitrogen, A11037	1:1000
Armenian Hamster IgG	Goat anti-Hamster IgG (H + L) Secondary Antibody [DyLight 488] (Pre-adsorbed), Novus Biologicals, NBP1-73008	1:1000
Rat IgG	Goat anti-Rat IgG (H + L) Cross-Adsorbed Secondary Antibody, Alexa Fluor™ 594, Invitrogen, A11007	1:800
Rat IgG	Goat anti-Rat IgG (H + L) Cross-Adsorbed Secondary Antibody, Alexa Fluor™ 680, Invitrogen, A21096	1:1000

### Immunohistochemistry and histopathology in sections and PCR

Excised globes were fixed in 2% PFA in 0.01 M PBS for 1 h at room temperature and then washed in 0.1 M TBS. The corneas were dissected and saturated stepwise through a gradient of 5%–20% sucrose in 0.1 M TBS as cryoprotectant to prevent cell deformation prior to embedding in Optimal Cutting Temperature compound (14-373–65, Thermo Fisher Scientific, Waltham, MA, United States) and freezing with liquid nitrogen. Samples were cut into 10 µm slices on a Leica CM3050 S cryostat and mounted onto Superfrost Plus^®^ slides. Thicker 50 µm sections were cut for immunohistochemistry for nerve stains.

For immunohistochemistry, sections were permeabilized in 0.3% Triton X-100 in 0.1 M TBS for 15 min, or with 1% Triton X-100 in 0.1 M TBS for thicker sections. They were then quenched for autofluorescence in 50 mM ammonium chloride for 30 min. Following that, they were blocked for 1 h in TBS containing 5% normal goat serum and 0.01 g/mL saponin, or in TBS containing 1% Triton X-100% and 5% FBS. Mouse on mouse blocking reagent was added to the blocking solution (Vector Laboratories, Burlingame, CA, United States) to reduce non-specific staining. Primary antibodies (Table 2) were diluted in the blocking solution and were added to the samples for an overnight incubation at 4°C. Sections were incubated for an hour, or 2 hours for thicker sections, with secondary antibodies (Table 2) which were diluted at 1:1000 in the blocking solution. Slides were quenched for autofluorescence using Vector TrueVIEW Autofluorescence Quenching Kit (Vector Laboratories, Burlingame, CA, United States). Nuclei were stained with 5 μg/mL DAPI for 10 min and mounted in Vectashield Vibrance Mounting Medium (Vector Laboratories, Burlingame, CA, United States).

All slides were imaged on a Zeiss LSM 880 confocal microscope (Zeiss, Oberkochen, Germany). For the exosomes immunolocalization, colocalization channels were built using minimum intensity thresholds of 13000 and 4500 for CD63 and TSG101, respectively. The exosome colocalization channel and Monocyte Chemoattractant Protein-1 (MCP-1) were combined the using minimum intensity thresholds of 5000 and 15000, respectively. For the nerve stains, images were taken on a Zeiss LSM 880 confocal microscope (Zeiss, Oberkochen, Germany) and processed using Imaris v9.1.2 software. Quantification was performed using Zen Blue v10.0.22000. Z-stacked images that were converted for maximum projection using extended depth of focus and the non-stained 488 channel was used to create a mask of the tissue. MFI of the tissue was calculated within the Zen Blue program. Expression of the Tac1 gene which encodes for Substance P was determined by quantitative PCR of Tac1 mRNA.

For hematoxylin and eosin (H&E) staining, the sections were rinsed in distilled water and stained with Harris hematoxylin (HHS32-1L, Sigma-Aldrich, St. Louis, MO, United States) for 8 min. Slides were washed and placed into a 1% acid alcohol solution and Scott’s tap water. Dehydration with 95% ethanol was done before staining with eosin-phloxine solution (HT110316-500ML, Sigma-Aldrich, St. Louis, MO, United States). All samples were cleared in xylene and mounted with Permount Mounting Medium (SP15-100, Thermo Fisher Scientific, Waltham, MA, United States) and imaged using Zeiss Axio Imager Z2 with an AxioCam MRc color CCD camera (Carl Zeiss, Oberkochen, Germany).

### Statistical analyses

Statistical analysis for BMDCs and flow cytometry expression of inflammatory markers were performed using GraphPad Prism and FlowJo. Aesthesiometry, fluorescein staining, OCT and body weights were analyzed using a two-way ANOVA with Tukey’s multiple comparisons test with a confidence interval of 95% for each group. Two-way ANOVA with Šidák’s multiple comparisons test was used to analyze tonometry, neovascularization, exosome expression and nerve expression.

## Results

### Micellar TA-A001

The average particle size of vehicle and TA-A001 micelles formed were respectively of 22.6 and 27.5 ± 1.3 nm with average particle distribution (PDI) of 0.11 and 0.07 ± 0.03. Encapsulation of TA-A001 within the micelles allowed for solutions of 0.125%, 0.25% and 0.5% to be prepared for testing.

### *In vitro* immune compatibility of TA-A001

**FIGURE 1:**
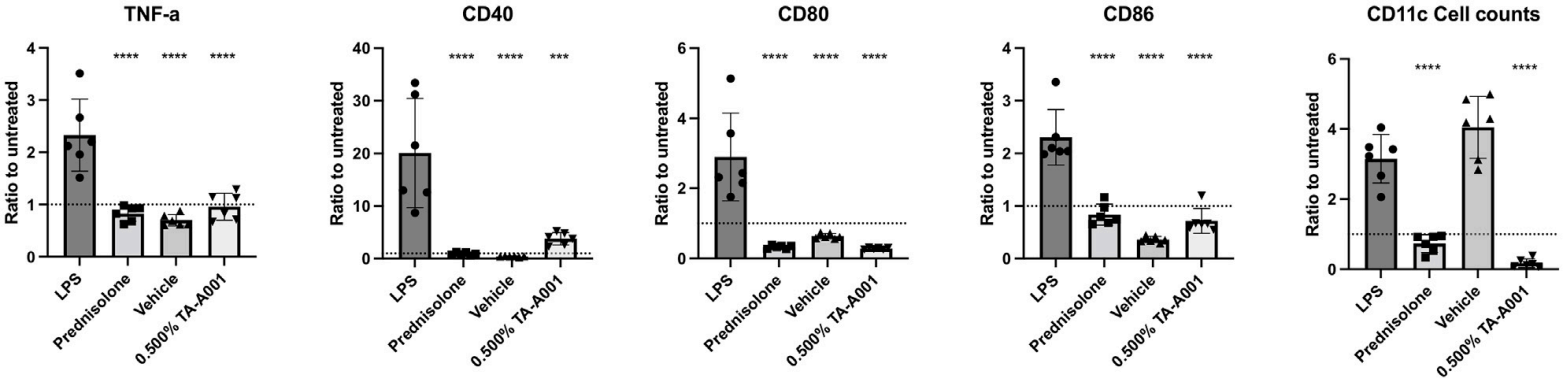
BMDC compatibility with CB2r agonist, TA-A001. BMDCs were treated with either prednisolone, SmartCelle delivery vehicle, or 0.500% TA-A001 delivered through SmartCelle. Flow cytometry was performed to measure the MFI of TNF-α, CD40, CD80, and CD86 as well as the counts of CD11^+^ live dendritic cells. Data was presented as a ratio to the untreated cells. Asterisks indicate significance compared to positive control, LPS. ***p ≤ 0.001 and ****p ≤ 0.0001 compared to LPS by one-way ANOVA Tukey’s test.

BMDC cultures were used to test the immune compatibility of CB2r agonist TA-A001, making sure that TA-A001 treatment does not activate dendritic cells, cells that can promote a pro-inflammatory response in the cornea and reduce visual acuity (43-47). Expression of immune markers CD40, CD80, CD86, and TNF-α were significantly lower for cells treated with prednisolone, 0.5% TA-A001 or SmartCelle delivery vehicle of TA-A001 only in comparison to cell treated with the positive control, LPS (Figure 1). As shown in Figure 1, TA-A001 does not activate dendritic cells.

### Clinical observations of corneal alkali burn treatments

**FIGURE 2:**
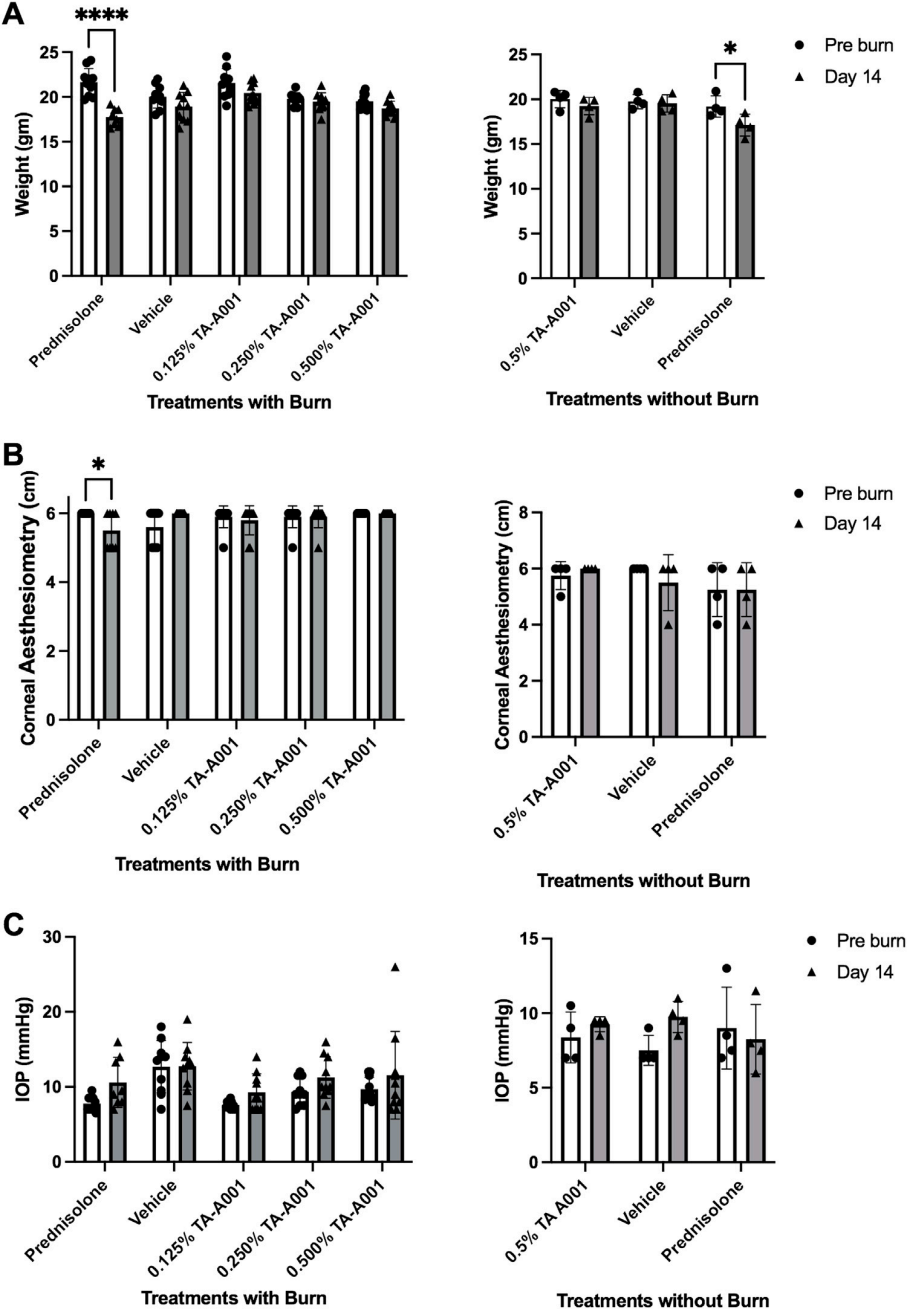
Clinical examination of mice treated with prednisolone, drug vehicle or different TA-A001 concentrations in alkali burned corneas and unburned, healthy corneas. Parameters monitored included **(A)** weight changes, **(B)** aesthesiometry measurements of touch sensitivity, and **(C)** Intraocular pressure (IOP) changes. *p ≤ 0.05, **p ≤ 0.01, ***p ≤ 0.001, and ****p ≤ 0.0001 by two-way ANOVA Tukey’s multiple comparisons test for **(A, B)**; tonometry measurements of IOP used two-way ANOVA Šidák’s multiple comparisons test.

Mice were observed for 14 days after the corneal alkali burn and subsequent treatment. Day 14 values are plotted for both with and without burn experiments. The weight of the mice of the prednisolone group gradually decreased over the 14 days, while such a variation was not observed in the other groups (Figure 2A). Loss of weight was even observed in the control experiment where treatment was administered without a burn. The group treated with prednisolone showed a significant drop in weight over the 14-day period. Aesthesiometry results showed that there was a decrease in sensation in the prednisolone group at 14 days post-treatment but no changes in the other groups (Figure 2B). No changes were observed between prednisolone and 0.5% TA-A001 in the group without burns. No significant increase in intraocular pressure (IOP) in any of the treatment groups observed (Figure 2C).

### Effect of TA-A001 treatment compared to steroid

**FIGURE 3:**
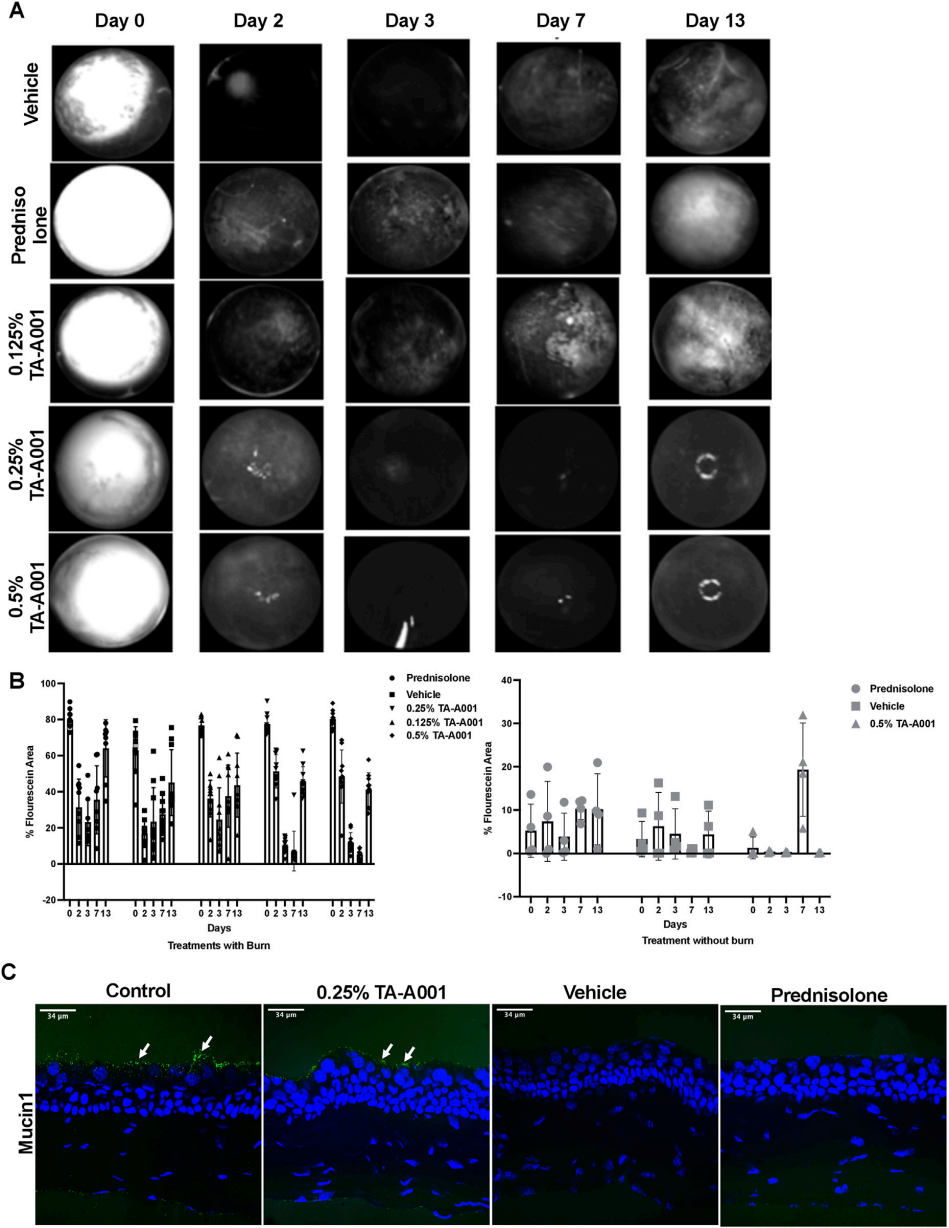
**(A)** Images of fluorescein staining showing wound closure rates of differentially treated corneas as bright patches. Fluorescein staining during re-epithelization is visible as diffuse haze. The rings of light in the 0.25% and 0.5% TA-A001 Day 13 images are reflection artifacts from the LED source. **(B)** Fluorescein staining quantification in corneas receiving treatments after burns and no burns. Grey symbols indicate diffuse fluorescein staining. **(C)** Mucin1 staining (arrows) on surface of the corneal epithelium (green fluorescence) of untreated controls and TA-A001-treated corneas. Scale bars, 34 µm.

Fluorescein exclusion by the epithelial cells was used to monitor the extent of epithelial injury and the rate of wound closure in response to the different treatments. On day 0, all the groups showed bright fluorescence due to the uptake of dye by the stroma indicating the extent of epithelial loss (Figure 3A). By day 2 of treatment, there was a significant decrease in the wound area in all the groups. However, between weeks one and two, the epithelia of prednisolone, vehicle and 0.125% TA-A001 treated corneas appear to be “leaky” as visualized by diffuse fluorescein staining. However, the fluorescein staining was more pronounced in the steroid and 0.125% TA-A001 treated corneas than the vehicle-treated corneas. These results are summarized in Figure 3B. When the drugs were administered to healthy corneas without burns, a diffuse haze of fluorescein staining was observed throughout the prednisolone treatment as well as vehicle, but in the TA-A001, it appeared around day 7 (Figure 3B). The corneal surface is protected by a layer of tear mucin. Staining with an antibody against Mucin1 showed that only the TA-A001 treated group had positive staining that was comparable to that seen in the untreated contralateral cornea (Figure 3C).

**FIGURE 4:**
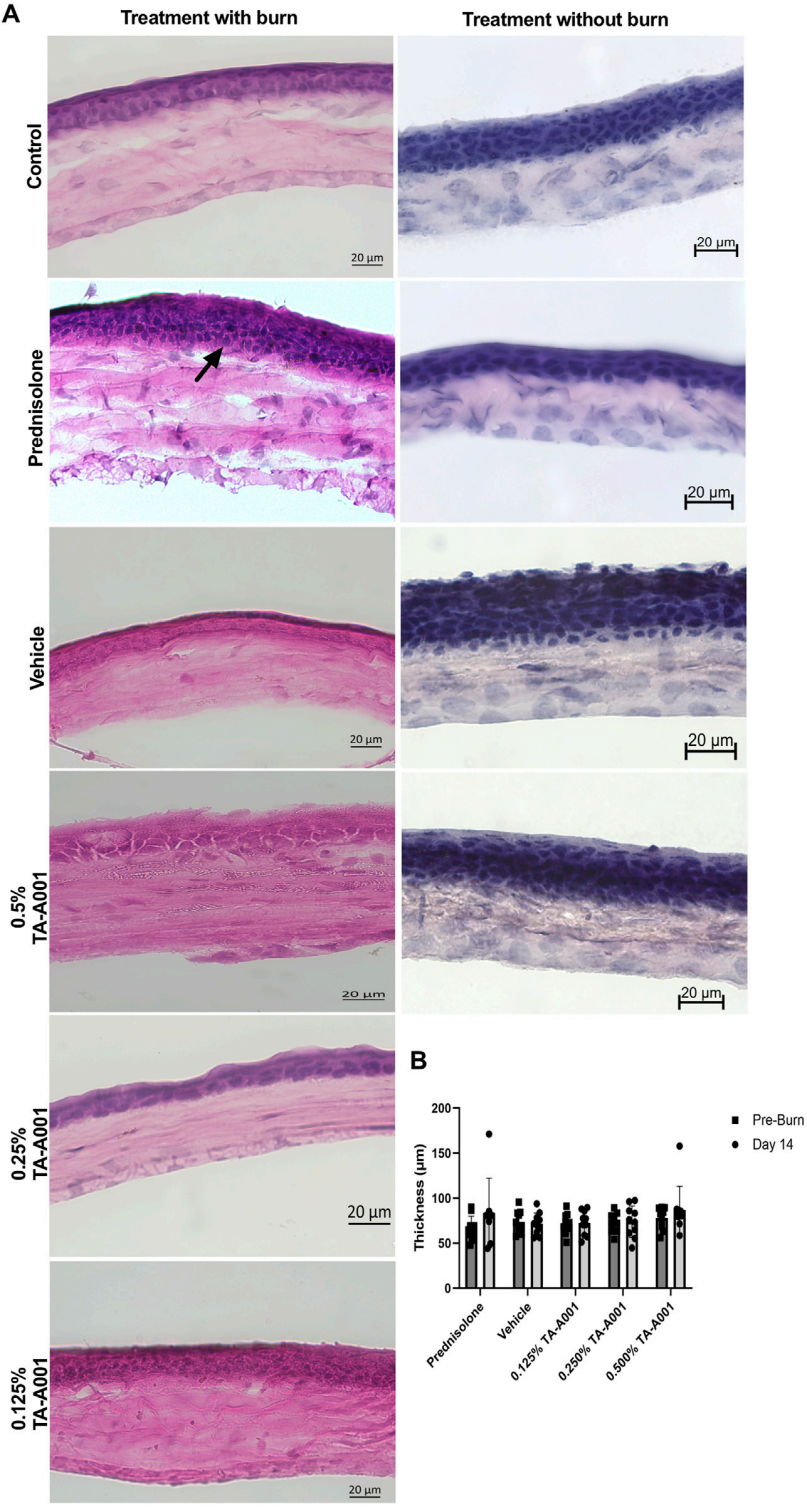
**(A)** H&E sections of alkali burned mouse corneas after 14 days post-treatment with TA-A001 compared to the drug vehicle and prednisolone. The arrow shows the change in thickness in the prednisolone-treated group compared to the others. Scale bars, 20 µm. **(B)** Thickness of corneas before and after alkali burn treatment as measured by optical coherence tomography.

Hematoxylin and Eosin (H&E) stained sections showed morphological differences in the different layers of the corneas of the different groups of mice (Figure 4A). Two mice of the prednisolone treated group showed an increase in thickness of the epithelium with a disorganised stromal layer, possibly due to inflammation or damage induced by the burn. The TA-A001 treated corneas on the other hand, showed a more even regeneration of the cornea, with an intact and regular epithelium and stromal layer. The thickness of the cornea measured using optical coherence tomography (OCT), however, did not show any significant differences amongst the groups (Figure 4B).

### Corneal neovascularization and inflammation post treatment

**FIGURE 5:**
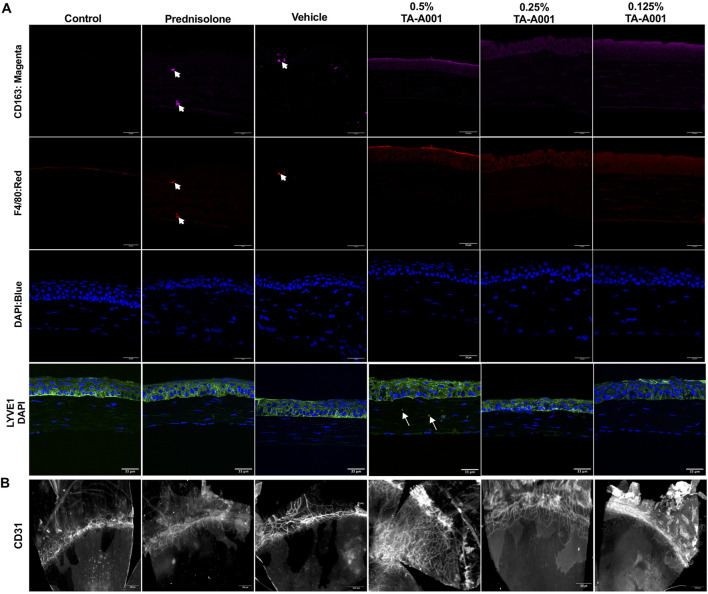
**(A)** Representative sections stained with antibodies against immune cells. Macrophages were stained with the F4/80 antibody (red), while M2 macrophages were localized by CD163 (magenta). LYVE1 staining (green) marks lymphatic vessels and macrophages. Blue, DAPI stained nuclei. Scale bars, 34 μm. **(B)** Flat mounts of alkali burned mouse corneas treated with different doses of TA-A001, drug vehicle or prednisolone for 14 days showing an increase in vascularization within the corneas of mice treated with the 0.5% dose of TA-A001. Scale bars, 300 µm.

Figure 5A shows F4/80 staining for macrophages in all the groups compared to the untreated controls. There was CD163 staining in the vehicle and TA-A001 groups. CD163 is a marker of anti-inflammatory M2 macrophages (Ly et al., 2010). LYVE1, which marks lymphatic vessels as well as macrophages (Lim et al., 2018) was seen in the epithelia of 0.25% TA-A001 and vehicle-treated corneas but only in the tear film region of prednisolone-treated corneas and those treated with 0.125% and 0.5% TA-A001.

Flat mount staining of blood vessels with anti-CD31 antibody showed that corneas treated with 0.5% TA-A001 were highly neovascularized (Figure 5B). The other groups had only had blood vessels in the areas adjacent to the normally avascular cornea. When the flat mounts were scored based on the level of neovascularization spread throughout the cornea and compared to levels in the contralateral untreated corneas the marked increase in neovascularization in the 0.5% TA-A001 group was observed. The difference between vascular ingrowth in the 0.5% and 0.125% TA-A001 groups was statistically significant, showing that the dose of drug received was an important factor in the treatment (Supplementary Figure S1).

### Extracellular vesicles secretion

**FIGURE 6:**
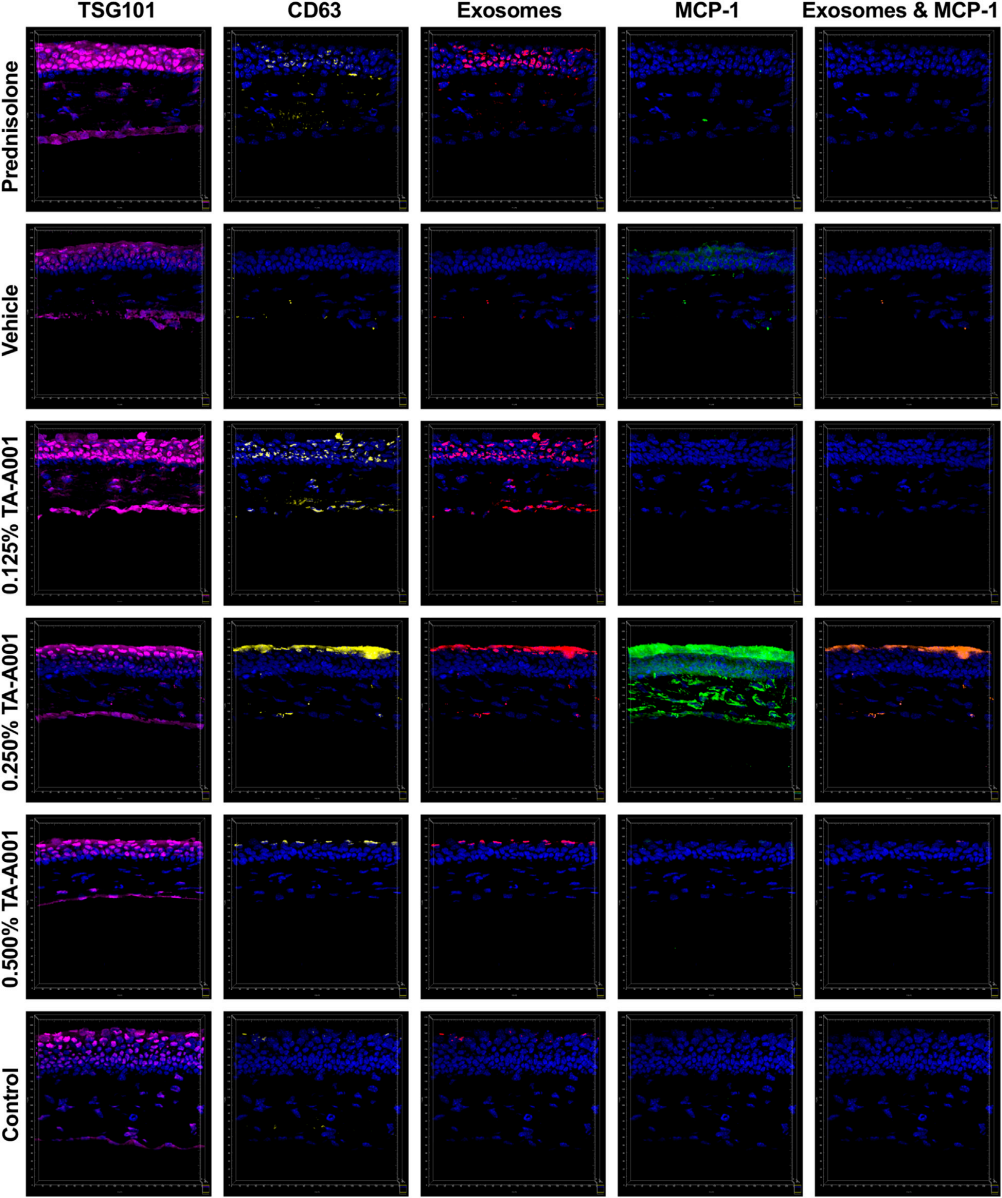
Secretion of extracellular vesicle markers, TSG101 (magenta) and CD63 (yellow), and monocyte chemoattractant protein-1 (MCP1; green) by alkali burned mouse corneas treated with one of prednisolone, drug vehicle or TA-A001. Colocalized channels of TSG101 and CD63 are indicators of exosomes (red). Brown staining shows colocalization of exosomes with MCP-1. All images were taken at the same magnification with scale bar representing 20 μm.

Alkali burned and treated corneas were evaluated for exosome secretion. Corneas treated with prednisolone, and 0.125% and 0.25% TA-A001 expressed TSG101 and CD63, markers of exosomes (Figure 6).

The corneas treated with 0.25% TA-A001 shows a higher MCP-1 expression especially within the stroma. Monocyte chemoattractant protein-1 (MCP-1) is a major cytokine that is involved in the recruitment of monocytes, but also plays an important role in endoplasmic reticulum stress. Damaged cell products could trigger an upregulation of MCP-1. Although MCP-1 expression was higher in the 0.25% TA-A001 group, this was not uniform in all the samples within the group.

### Corneal nerves

**FIGURE 7:**
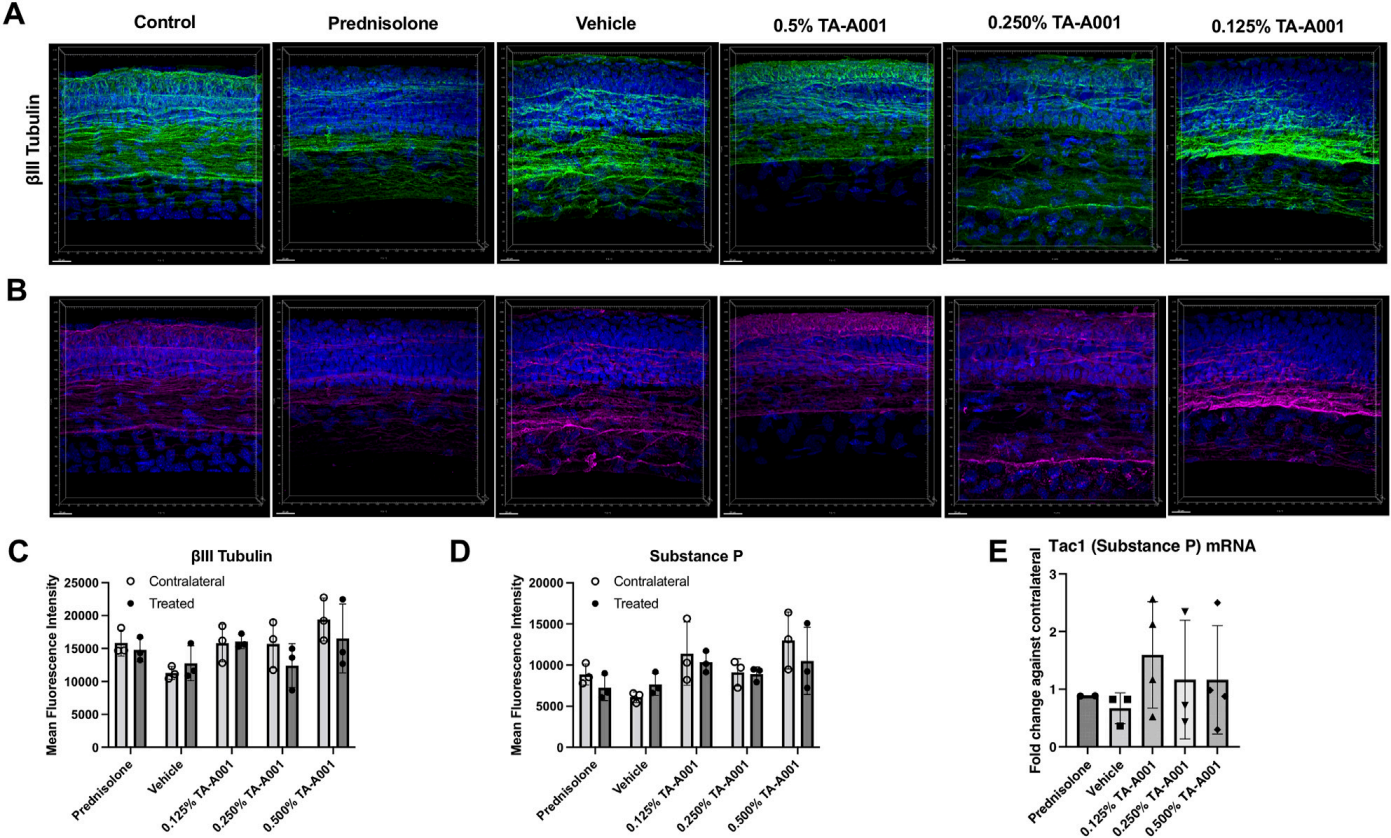
Corneal nerve density and morphology after prednisolone, drug vehicle or TA-A001 treatment. **(A, B)** Thick cryosections were stained for βIII tubulin (green), Substance P (magenta), and DAPI (blue), *n* = three mice per group. Quantification of βIII tubulin **(C)** and substance P **(D)** as determined by measuring mean fluorescence intensity (MFI). **(E)** mRNA expression of Tac1 of mouse eyes as determined by qPCR. N = 3 samples per group. Scale bar, 20 µm.

The cornea is supplied by sensory nerves from the trigeminal ganglia. βIII tubulin is a structural protein within the microtubules of neurites. βIII tubulin-positive sensory fibres were found throughout the epithelium of TA-A001-treated corneas, including the more superficial layers, particularly in the 0.25% and 0.5% doses (Figure 7A). These staining patterns were similar to that of the untreated, healthy control eyes. Vehicle treated corneas did not have nerve fibres in the superficial layers. Prednisolone treated corneas showed fewer stained nerve fibres, and in particular, they were absent from the superficial layers. Quantification of mean fluorescence intensity from the immunohistochemistry, however, did not show any significant differences between treated corneas and their contralateral untreated controls, with *n* = 3 samples per group (Figure 7C).

Staining with an antibody against Substance P, a pro-inflammatory neuropeptide, revealed slightly different expressions among the groups. Substance P levels were lower in prednisolone treated mice compared to the TA-A001 and vehicle treated mice (Figure 7B), although the difference was not significant (*p* > 0.05) (Figure 7D). As with βIII tubulin staining, Substance P positive nerve fibres in the 0.25% and 0.5% TA-A001 groups extended to the superficial layers of the corneal epithelium, like in the control untreated contralateral corneas. The mRNA expression of Substance P was similar to those of their untreated contralateral corneas (Figure 7E).

## Discussion

Corticosteroids are often prescribed to patients suffering from corneal pain and inflammation in the clinic. While effective at reducing inflammation, they also come with notable side effects, in particular, elevated IOP and glaucoma (Kersey and Broadway, 2006). Agonists to cannabinoids receptors (CBr) are a potential alternative treatment, with publications showcasing the improved wound healing and decreased IOP with CBr agonist treatment (Yang et al., 2013; Cairns et al., 2016; Murataeva et al., 2019; Steven et al., 2019). Here, alkali burns were used to induce corneal inflammation in mice, which were then treated with one of three concentrations of TA-A001, the vehicle alone, or prednisolone. The alkali burns induced with 0.25N NaOH were moderate, designed to simulate the level of inflammation and pain observed in patients seen in the clinic with keratitis. These burns did not result in stromal melting or thinning that is often associated with severe burns. This study aims to compare the safety and efficacy of micellar TA-A001 as a potential alternative to corticosteroids for treating moderate chemical burns.

CB2r agonist TA-A001 delivered through the SmartCelle vehicle that enabled its uptake within aqueous environment, the vehicle alone, and prednisolone were tested. These treatments were given to BMDCs. The expression of inflammatory markers TNF-α, CD80, and CD86 were similar between TA-A001 treated cells and those treated with prednisolone. CD40 was expressed slightly more in TA-A001 treated cells but were still significantly lower than the cells treated with the positive control, LPS. Since the CD40 expression was still very low compared to the LPS activated cells, the CD40 may not be playing a pro-inflammatory role. Low levels of CD40 on dendritic cells can promote regulatory T cells generation (Martin et al., 2010). Additionally, BMDC counts were very low in TA-A001 treated cultures which coincides with previous studies claiming that CBrs can promote NF-κB-dependent apoptosis (Do et al., 2004). The combination of causing apoptosis and low expression of CD40 in BMDCs may play a role in reducing inflammation in a live model.

Mice treated with prednisolone had eyes that were less touch sensitive and had more diffuse fluorescein staining at 2 weeks. They also had a higher IOP than before the treatment, although not statistically significant. Importantly, lowering the IOP reduces the risk of developing glaucoma (Jayaram, 2020). The clinical results show that while prednisolone treats the burns, it does have a negative impact on not just the cornea but the overall health of the mouse. CB may be associated with increased remodeling and extracellular matrix degradation in the trabecular meshwork, leading to lower IOP. Corneal melting, a potential complication of alkali burn whose pathogenesis also involves extracellular matrix degradation by matrix metalloproteinase (Dohlman et al., 2018; Dohlman et al., 2019) was not seen. The absence of keratolysis in our study is an important safety feature for this group of drugs. Further, the prednisolone treated mice lost weight over the 2-week study that indicated an adverse effect on overall health of the animals, which was not observed in the other groups. This weight loss may be related to increased corneal pain or discomfort in this group. On the contrary, mice treated either 0.250% TA-A001 or 0.500% TA-A001 had no weight loss (Figure 2). The mice treated with higher concentrations of TA-A001 performed much better in the clinical examinations compared to the prednisolone treated mice.

Post-mortem immunohistochemical examinations of sectioned corneas were performed to obtain a more complete understanding of what is occurring in these mice. Neovascularization was measured in the mice by staining for CD31, a marker that can stain blood vessels in the cornea (Le et al., 2018). The mice treated with 0.500% TA-A001 had a significantly more vasculature expression in the cornea. This coincides with a previous cannabinoid study where a CB1r blockade reduced neovascularization in the cornea (Pisanti et al., 2011). In addition to being associated with inflammation, neovascularization in the cornea often causes a decrease in vision and optical clarity, suggesting that care is needed when determining the optimal dosage of TA-A001 (Clements and Dana, 2011; Sharif and Sharif, 2019). Of note, no corneal vascularization was noted in the central cornea, where it could potentially impact vision. Subtle increases in peripheral vascularization may be helpful in the subacute healing phase of a corneal burn (Conn et al., 1980), particularly if inflammatory markers are not significantly increased.

To better understand cell-cell communication during inflammation resulting from a corneal alkali burn, we examined the role of exosomes, which have been shown to be involved in normal as well as disease states in various tissues. We examined the expression of exosomes markers TSG101 and CD63, as well as MCP-1, a marker for inflammation and a potent chemoattractant for monocytes/macrophages that has been found within exosomes (Wang et al., 2023). In the epithelium, there was a significant increase of potentially anti-inflammatory M2 CD63-positive macrophages together with the increased MCP-1 expression for mice treated with 0.250% TA-A001 while the other treatments did not have a significant difference. MCP-1 has been shown to be colocalized with exosomes. Interestingly, CD63 and MCP-1 had very low expression in the 0.500% treated mice despite these mice having more corneal neovascularization. Corneas treated with 0.5% TA-A001 did not show any increase in CD163 either. This could explain why the 0.500% TA-A001 treated mice had a significant increase in neovascularization with low MCP-1 expression, since there was no increase in M2 macrophages in these corneas, leading to the neovascularization observed at day 14. Unfortunately, MCP-1 expression was only examined at day 14, requiring additional timepoints to study if the time of high MCP-1 expression was dose dependent.

Substance P is present in corneal nerves and contributes to the ocular inflammatory response. The neuropeptide is stored within neurons in the cornea and upon stress, is released to promote inflammation through stimulating macrophages and promoting vascularization (Hoyer and Bartfai, 2012; Bignami et al., 2016; Byun et al., 2020; Liu et al., 2020). Staining for βIII tubulin, a nerve marker, and Substance P showed a non-significant increase in Substance P expression with TA-A001 treatment. Interestingly, in the images, Substance P was highly colocalized with βIII tubulin, suggesting that Substance P was not released and remained within the nerves. This observation is further supported by observations that corneal trigeminal neurons do not normally express Substance P even under inflammatory conditions, even though this can be triggered experimentally with the insignificant difference in Tac1 mRNA expression within the eye. Under inflammatory conditions (Byun et al., 2020), increased expression of Tac1 mRNA can be found in the cornea, so the lack of Tac1 expression seen in TA-A001 treated mice suggests the lack of production and release of Substance P. Since Substance P remained in the nerves and that Tac1 expression did not significantly differ, it is unlikely that Substance P contributed to the neovascularization seen in the 0.500% TA-A001 treated mice. Additionally, when observing βIII tubulin staining, the innervation of the epithelium appears slightly different between the treatments. The epithelial innervation of 0.500% TA-A001 treated mice were like the non-burned contralateral eyes, while prednisolone treated mice had hazier and more diffuse βIII tubulin staining. This suggests that TA-A001 treatment was able to help sensory neuron regrowth following the alkali burn and is probably why only the prednisolone treated mice showed a decrease in corneal sensitivity.

We have shown that CBr2 agonist TA-A001 delivered within micelles showed dose-dependent results. The 0.25% TA-A001 dose appeared to produce optimal wound healing results. With further testing, TA-A001 might prove to be a potential alternative to corticosteroids for treating corneal inflammation caused by alkali burns or other causes.

## Data Availability

The original contributions presented in the study are included in the article/Supplementary Material, further inquiries can be directed to the corresponding authors.
