# Supplementary material for: Novel micellar CB2 receptor agonist with anti-inflammatory action for treating corneal alkali burns in a mouse model
Source: Front Pharmacol. 2023 Dec 15;14:1270699. doi: 10.3389/fphar.2023.1270699 (PMC10755873; doi:10.3389/fphar.2023.1270699)
Supplement: Supplementary file 1 [file DataSheet1.PDF]

Supplementary Material

Novel micellar CB2 receptor agonist with anti-inflammatory action for treating corneal alkali burns in a mouse model

Neethi C. Thathapudi[1,2,3,§], Marc Groleau[1,2,4,§], Delali S. Degue[1,2,3], Mozhgan Aghajanzadeh Kiyaseh[1,2,3], Piotr Kujawa[5], Fouzia Soulhi[5], Naoufal Akla[1,2], May Griffith[1,2,3,*], Marie-Claude Robert[1,2,3,*]

[1]Maisonneuve-Rosemont Hospital Research Centre, Montreal, Quebec, Canada
[2]Department of Ophthalmology, Université de Montréal, Montreal, Quebec, Canada
[3]Institute of Biomedical Engineering, Université de Montréal, Montreal, Quebec, Canada
[4]Department of Microbiology, Infectiology and Immunology, Université de Montréal, Montreal, Quebec, Canada
[5]Pharmaceutical Research and Development, Altus Formulation Inc, Laval, Quebec, Canada

* Corresponding Author: marie-claude.robert.2@umontreal.ca, may.griffith@umontreal.ca

Details of SmartCelle Synthesis

Briefly, PLA-OH was synthesized via the ROP of D,L – lactide (recrystallized rom ethyl acetate before use) using diethylene glycol monoethyl ether (DEGMEE) as initiator and Sn (Oct) 2 as the catalyst under an argon atmosphere. The reaction mixture was heated at 150°C for 24 hrs. After cooling to room temperature, the crude product obtained was dissolved in certain quantity of DCM and precipitated from hexanes. The precipitated polymer was collected by filtration and then redissolved in DCM followed by precipitation from hexanes once again. The collected PLA -OH was dried under vacuum at 40°C. Subsequently PLA-OH was dissolved in THF with triethylamine while stirring under argon atmosphere. The reaction mixture was cooled in an ice bath. After the cooling, 2 - bromopropionyl bromide was added drop wise. The reaction mixture was then stirred for 72 hrs at room temperature. The precipitated trimethylamine salt was removed by filtration and the filtrate was evaporated until dryness. The solid product was dissolved in DCM and washed methodically with saturated sodium bicarbonate solution. The organic layer was washed with water, dried over anhydrous sodium sulphate and filtered. The filtrate was concentrated by evaporation and PLA-BR precipitated from hexanes and dried under vacuum at 40°C. Subsequently the prepared PLA - Br was mixed with potassium ethyl xanthogenate (POEX) in DCM and the mixture was purged with argon during 2 hrs. Pyridine was mixed with DCM and then was added to the reaction mixture of PLA-Br. The reaction mixture was stirred at room temperature for 48 hrs. The reaction solution was washed consecutively with saturated ammonium chloride solution and saturated sodium bicarbonate solution. The organic layer was washed with more water, dried over anhydrous sodium sulphate, and filtered. The PLA - POEX filtrate was concentrated by evaporation and precipitated from hexanes and dried under vacuum

at 40°C. Subsequently PLA - POEX was dissolved in THF. To this solution N-vinyl-2-pyrrolidone (NVP) and azobisisobutyronitrile (AIBN) were added and dissolved. The solution was continuously stirred for 48 hrs under argon atmosphere at 80°C. The crude PVP-PLA was dissolved in DCM, precipitated from hexanes and dried under vacuum at 40 ° C. The polymer was purified more by repeated the dissolution in DCM and precipitated from hexanes and finally dried under vacuum at 40 ° C. Block copolymers used in this study had a molecular weight of 5736 g/mol with a PVP:PLA block ratio of 30:29.

Supplementary Figure

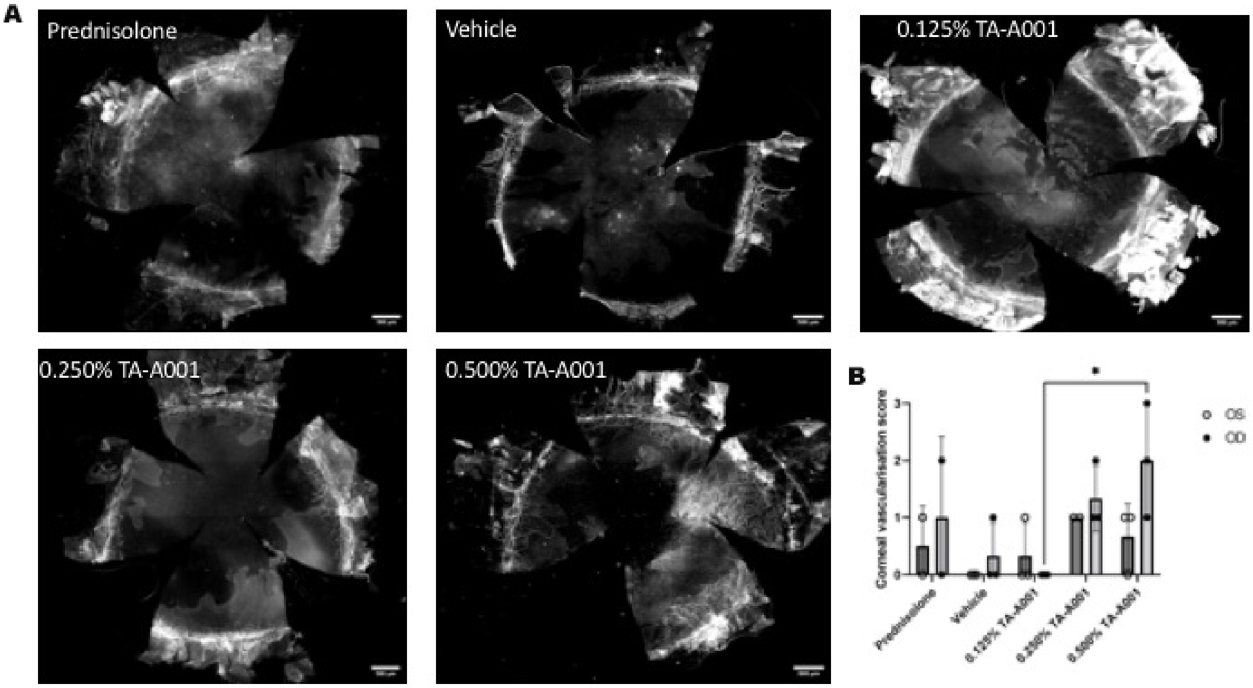

Figure S1. A) Flat mounts of alkali burned mouse corneas treated with different doses of TA-A001, drug vehicle or prednisolone for 14 days. An increase in vascular ingrowth towards the centre of the cornea was seen in mice treated with the 0.5% dose of TA-A001. Scale bars, 500 µm. B) Graph showing the extent of corneal neovascularization scored from 0-3, where 0 = < 300µm of vessel growth towards the central cornea, 1 = 300-400 µm, 2 = 401-500 µm, 3 = >500 µm. *P≤0.05 by two-way ANOVA Šidák's multiple comparisons test.
